# PCR arrays identify metallothionein-3 as a highly hypoxia-inducible gene in human adipocytes

**DOI:** 10.1016/j.bbrc.2008.01.036

**Published:** 2008-03-28

**Authors:** Bohan Wang, I. Stuart Wood, Paul Trayhurn

**Affiliations:** Obesity Biology Unit, School of Clinical Sciences, University of Liverpool, Duncan Building, Liverpool L69 3GA, UK

**Keywords:** Adipocyte, Adipokine, Adipose tissue, HIF-1, Gene expression, Hypoxia, Metallothionein, Obesity, Oxygen tension, PCR arrays

## Abstract

Hypoxia-signalling pathway PCR arrays were used to examine the integrated response of human adipocytes to low O_2_ tension. Incubation of adipocytes in 1% O_2_ for 24 h resulted in no change in the expression of 63 of the 84 genes on the arrays, a reduction in expression of 9 genes (including uncoupling protein 2) and increased expression of 12 genes. Substantial increases (>10-fold) in leptin, angiopoietin-like protein 4, VEGF and GLUT-1 mRNA levels were observed. The expression of one gene, metallothionein-3 (MT-3), was dramatically (>600-fold) and rapidly (by 60 min) increased by hypoxia. MT-3 gene expression was also substantially induced by hypoxia mimetics (CoCl_2_, desferrioxamine, dimethyloxalylglycine), indicating transcriptional regulation through HIF-1. Hypoxia additionally induced MT-3 expression in preadipocytes, and MT-3 mRNA was detected in human (obese) subcutaneous and omental adipose tissue. MT-3 is a highly hypoxia-inducible gene in human adipocytes; the protein may protect adipocytes from hypoxic damage.

White adipose tissue (WAT) is now recognised as a key endocrine organ with white adipocytes secreting a multiplicity of protein hormones and factors [1–4]. A number of these adipokines are linked to immunity and inflammation, and a major inflammatory response is evident in WAT in obesity [3,5,6]. This state of inflammation is linked to several of the diseases associated with the obese state, particularly type 2 diabetes and the metabolic syndrome [4,6,7]. We have proposed that inflammation in adipose tissue in obesity is primarily a consequence of hypoxia in clusters of adipocytes that become distant from the vasculature as tissue mass expands [3,8]. It has also been suggested that inflammation may be a response to endoplasmic reticulum stress, or to reactive oxygen species [9–11].

Recent studies on *ob/ob*, KKAy and diet-induced obese mice have provided evidence that hypoxia occurs in WAT in obesity [12–14]. Elevated levels of the hypoxia-inducible transcription factor subunit, HIF-1α, together with increased expression of hypoxia-sensitive genes, such as leptin, in obese mice are also consistent with low O_2_ tension in WAT in obesity [12–14]. Cell culture studies have directly demonstrated that the expression of several genes encoding inflammation-related adipokines, including leptin, PAI-I, VEGF and visfatin, are hypoxia-inducible in murine adipocytes [12,13,15–17]. Production of adiponectin, which has an anti-inflammatory action [18,19], is suppressed, however, by hypoxia [16,20]. Hypoxia has further been shown to modulate the expression of inflammation-related adipokine genes in human adipocytes, particularly leptin, angiopoietin-like protein 4 and VEGF [20]. The expression of facilitative glucose transporters, notably GLUT-1, is also induced by low O_2_ tension in human adipocytes, and this is accompanied by a parallel increase in glucose uptake [21].

Both the murine and human adipocyte cell culture studies have employed a selective ‘candidate gene’ approach. To obtain a more global view of hypoxia-sensitive gene expression in human adipocytes we have utilised hypoxia-signalling pathway PCR arrays. Application of the arrays has identified a member of the metallothionein family of low mol. wt., cysteine-rich, proteins, metallothionein-3 (MT-3), as a highly hypoxia-inducible gene in human adipocytes. We show that expression of the MT-3 gene is rapidly and dramatically induced in human adipocytes by hypoxia.

## Materials and methods

*Human adipose tissue and adipocyte cell culture.* Subcutaneous and omental WAT was obtained from 3 (1 male, 2 female) obese subjects (BMI 43 ± 6) undergoing gastroplasty (provided by Dr John Pinkney). Ethical permission was obtained through the Sefton Ethics Committee; the subjects gave informed consent for the removal of fat samples.

Human subcutaneous preadipocytes were obtained, with culture media, from Zen-Bio (USA) and cultured as previously [20]. The cells were from patients with a mean BMI of 25 (average age 41 years). Cells were trypsinized from a 75 cm^2^ flask and plated at 40,000/cm^2^ in a 24-well plate and maintained in preadipocyte medium containing DMEM/Ham’s F12 (1:1, v/v), 10% fetal calf serum (FCS), 15 mM HEPES, 100 U/ml penicillin, 100 μg/ml streptomycin, and 0.25 μg/ml amphotericin B at 37 °C in a humidified atmosphere of 95% air/5% CO_2_. Cells were induced at confluence in differentiation medium (DM) composed of adipose medium (AM) supplemented with 0.25 mM isobutyl methylxanthine (IBMX) and 10 μM of a PPARγ agonist for 5 days. The cells were cultured with AM containing DMEM/Ham’s F-12 (1:1, v/v), 3% FCS, 1 μM dexamethasone, 100 U/ml penicillin, 100 μg/ml streptomycin, and 0.25 μg/ml amphotericin B. The medium was changed every 3 days.

Some preadipocytes were collected at the induction day (day 0), divided into two groups and incubated under 1% O_2_ or normoxia (21% O_2_) for 24 h. Fully differentiated cells at day 15 post-induction were treated with hypoxia mimetics or exposed to 1% O_2_ for up to 24 h. For chemical hypoxia, 100 μM CoCl_2_, 150 μM desferrioxamine or 500 μM dimethyloxalylglycine was added to the wells. For exposure to hypoxia, the cells were transferred to a MIC-101 modular incubator chamber (Billups-Rosenberg, USA), which was flushed with 1% O_2_/94% N_2_/5% CO_2_, sealed, and placed at 37 °C for up to 24 h. Control cells were cultured in a standard incubator (21% O_2_/5% CO_2_). Cells were harvested in Trizol at the stated time-points. All incubations were in replicates of up to 6 wells.

*RNA extraction and cDNA synthesis.* Total RNA was isolated using Trizol, and 1 μg reverse transcribed with a Reverse-iT™ 1^ST^ Strand Synthesis Kit (Abgene, UK) in the presence of anchored oligo dT.

*PCR arrays.* Human hypoxia signalling pathway RT^2^ Profiler™ PCR Arrays (SuperArray, USA) were used to screen the expression of multiple genes (total 84) in human adipocytes exposed to 1% O_2_. These arrays combine the ability to simultaneously screen a number of pathway genes with the quantitative precision of real-time PCR, using SYBR Green technology. They were used according to the manufacturer’s protocol on a Stratagene Mx3005P detector.

*RT-PCR.* MT-3 primers were synthesized commercially (Eurogentec, UK): (173 bp product), 5′-AGACCTGCCCCTGCCCTTC-3′ (forward), 5′-TGCTTCTGCCTCAGCTGCC-3′ (reverse). RT-PCR was performed with 2 μl of RT cDNA and specific primers (0.2 μM of forward and 0.2 μM of reverse) and 1.1× Ready Mix™ PCR Master Mix (Abgene) in a volume of 25 μl. PCR conditions were: 2 min at 94 °C for denaturation followed by 32 cycles for MT-3 and 23 cycles for β-actin of 20 s at 94 °C for denaturation, 30 s at the optimal annealing temperature and 45 s at 72 °C for extension, with a final elongation step of 10 min at 72 °C. Negative controls were performed without templates. PCR products were sequenced (Eurogentec) to confirm their identity.

*Real-time PCR.* Real-time PCR reactions were carried out in a final volume of 12.5 μl consisting of 12.5–50 ng of reverse transcribed cDNA with optimal concentrations of primers and probe and qPCR™ Core kit (Eurogentec) in 96-well plates on a Stratagene Mx3005P detector. Primer and probe sets were synthesized commercially (Eurogentec). *Taq*Man probes were labelled with a reporter fluorescent dye (FAM: 6-carboxyfluorescein) at the 5′-end and a fluorescent dye quencher (TAMRA: 6-carboxy-tetramethyl-rhodamine) at the 3′-end. The sequence and concentrations of primers and probes, together with the product size were as previously [20,22,23], except for MT-3. The MT-3 sequences (GenBank Accession No. NM_005954) were: 5′-CTGCCCCTGCCCTTCTGG-3′ (forward), 5′-TCTCCGCCTTTGCACACAC-3′ (reverse), and 5′-FAM-CTTGGCACACTTCTCACACTCCGCAGG-TAMRA-3′ (probe). Typically, the amplification started with 2 min at 50 °C, 10 min at 95 °C, and then 40 cycles of the following: 15 s at 95 °C and 1 min at 60 °C.

Human POLR2A was used as reference. Relative quantitation values were expressed using the 2^−ΔΔCt^ method (User bulletin #2, ABI Prism 7700, Applied Biosystems) as fold changes in the target gene normalized to the reference gene and related to the expression of the controls. PCR efficiency was close to 100% and samples were analyzed in at least duplicate.

*Statistical analysis.* Differences between groups were analyzed by an unpaired Student’s *t-*test.

## Results

### PCR arrays

In the first experiments, human adipocytes differentiated from preadipocytes in culture were incubated for 24 h under either hypoxic conditions (1% O_2_) or normoxia (21% O_2_). The cells were screened using hypoxia-signalling pathway PCR arrays. Of the 84 hypoxia-sensitive genes in the arrays, the mRNA level of 63 (75%) did not significantly change (threshold of >2-fold change at *P* < 0.05) with hypoxia. These genes included IL-1α, PPARα and GLUT-4. A significant reduction in mRNA level was observed with 9 genes (11%). The largest reduction (>4-fold) was for caspase 1 and HIF-1α, with smaller (2- to 3-fold) falls for the remaining 7 genes, which included glutathione peroxidase 1, catalase and uncoupling protein 2 (Table 1).

The mRNA level of a total of 12 genes (14%) was upregulated by hypoxia (Table 1). These included leptin (20.9-fold increase), VEGF (20.7-fold increase), angiopoietin-like protein 4 (10.5-fold increase), IL-6 (2.9-fold increase) and GLUT-1 (12.4-fold increase). Other genes which were upregulated by hypoxia included cytoglobin, IGF-2 and glucose phosphate isomerase; the level of the mRNA encoding these genes increased 2- to 4-fold in hypoxia. One particular gene, metallothionein-3 (MT-3), showed a dramatic increase in expression in response to hypoxia, the mRNA level rising by >600-fold.

The acute response to hypoxia was then examined in cells exposed to low O_2_ tension for 4 rather than 24 h. No significant change in mRNA level was evident with 76 of the 84 genes (90%) on the arrays, and no gene showed a significant reduction in mRNA. There was, however, a significant increase in expression with 8 genes (9.5%) and 5 of these, including GLUT-1, VEGF and leptin, were increased at both 4 and 24 h. The largest increase was again with MT-3, the mRNA level being 121-fold higher than in normoxic cells.

### Metallothionein-3 gene expression

**Fig. 1:**
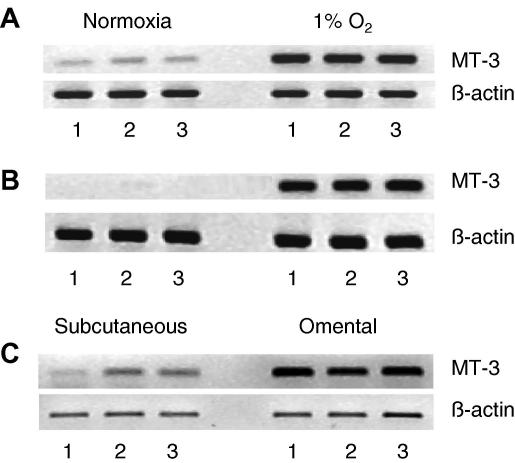
MT-3 gene expression in human adipocytes and adipose tissue by RT-PCR. (A) Effect of hypoxia (1% O_2_ for 24 h) on MT-3 gene expression in human adipocytes differentiated in culture. (B) Effect of hypoxia on MT-3 gene expression in human preadipocytes. Control cells were incubated under normoxia (21% O_2_). Representative gels for three sets of cells are shown. (C) MT-3 gene expression in human subcutaneous and omental adipose tissue from three obese subjects.

To substantiate the observation from the arrays that the MT-3 gene is strongly induced in human adipocytes by hypoxia, RT-PCR was performed using specific primers. Only a faint band was observed in normoxia, while a strong band of the size expected for MT-3 was observed with cells exposed to hypoxia for 24 h (Fig. 1A). Sequence analysis confirmed that the band corresponded to human MT-3 mRNA.

MT-3 gene expression was then examined by RT-PCR in preadipocytes. MT-3 mRNA was not detected in preadipocytes maintained in normoxia, but a strong signal was obtained in those incubated in 1% O_2_ (Fig. 1B). To determine whether the MT-3 gene is expressed in adipose tissue *in vivo*, subcutaneous and omental fat from three obese patients was examined. MT-3 mRNA was detected by RT-PCR in both adipose tissue depots, the signal being strongest in omental fat (Fig. 1C).

**Fig. 2:**
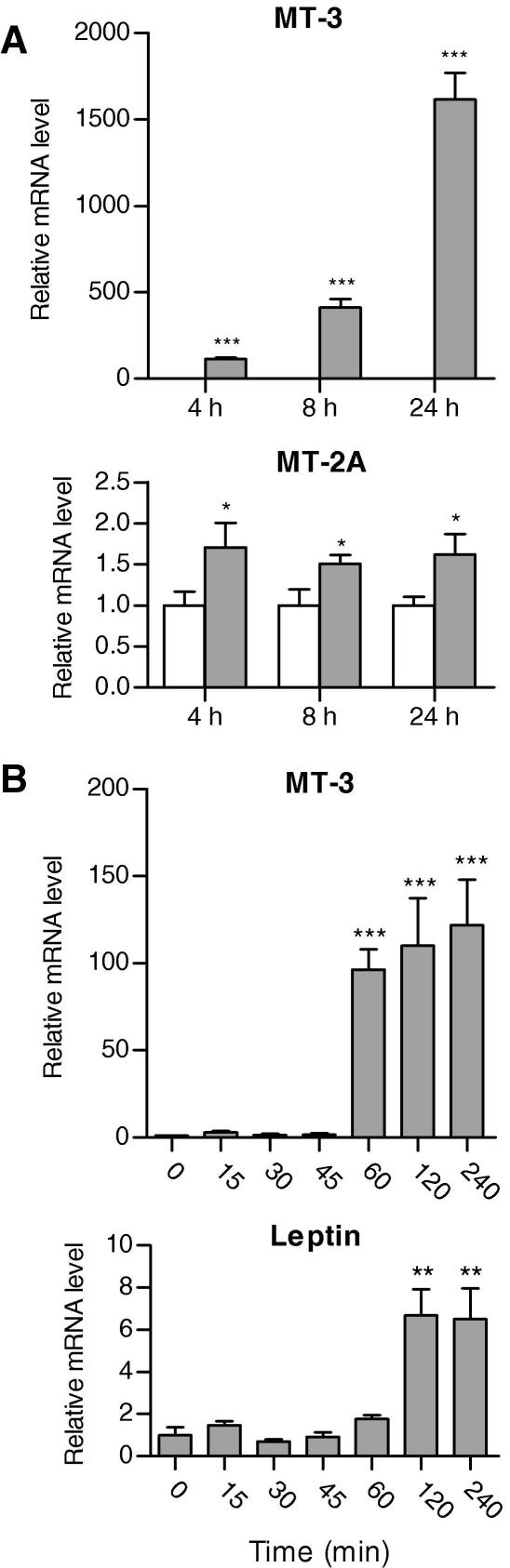
Time-course of hypoxia-induced MT-3 gene expression in human adipocytes by real-time PCR. Human adipocytes, differentiated in culture, were incubated under normoxia (21% O_2_) or hypoxia (1% O_2_) for the stated times. (A) MT-3 and MT-2A mRNA levels relative to normoxic controls at 4, 8, and 24 h of hypoxia. (B) Effect of acute exposure to hypoxia on MT-3 and leptin mRNA levels. Results are mean values ± SE (bars) for 4 groups of adipocytes. *^∗^P* < 0.05*; ^∗∗^P* *<* 0.01; *^∗∗∗^P* < 0.001, compared with normoxia.

The response to hypoxia of MT-2A, another member of the metallothionein family expressed in human adipocytes [24], was next compared by real-time PCR using Taqman probes. MT-3 mRNA level was increased, using this approach, by 112-fold at 4 h and >1600-fold at 24 h (Fig. 2A). However, there was minimal change (<2-fold increase) in MT-2A mRNA level at 4–24 h of exposure to hypoxia (Fig. 2A).

In view of the substantial increase in MT-3 mRNA following 4 h under hypoxic conditions, a detailed time-course of the early response to low O_2_ tension was examined. During the first 45 min in 1% O_2_, MT-3 mRNA level remained similar to that of cells under normoxia, but a major increase occurred between 45 and 60 min; by 60 min MT-3 mRNA increased almost 100-fold and this increase was sustained over the following 3 h (Fig 2B). Leptin mRNA level, measured for comparison, changed later, at between 60 and 120 min (Fig. 2B).

**Fig. 3:**
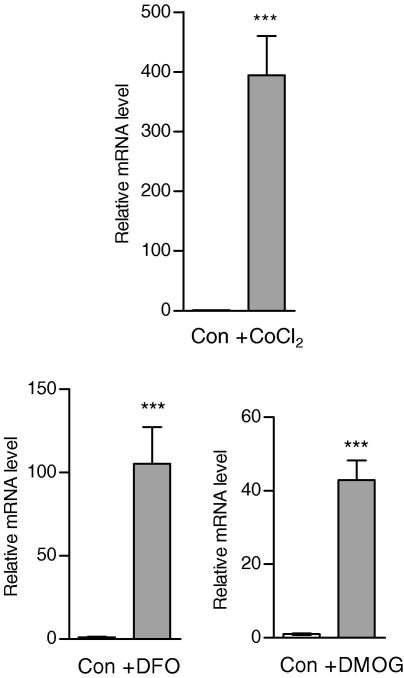
Effect of hypoxia mimetics on MT-3 gene expression in human adipocytes. Human adipocytes, differentiated in culture, were incubated with CoCl_2_ (100 μM), desferrioxamine (DFO; 150 μM) or dimethyloxalylglycine (DMOG; 500 μM) for 24 h; control adipocytes (Con) received no addition. MT-3 mRNA levels were measured by real-time PCR. Results are mean values ± SE (bars) for 4–6 groups of adipocytes. *^∗∗∗^P* < 0.001, compared with control cells.

In the final experiments, the effects of chemically induced hypoxia on MT-3 gene expression were examined. Three different mimetics were used (CoCl_2_, desferrioxamine, dimethyloxalylglycine), each of which leads to the stabilisation of HIF-1α. Incubation with each hypoxia mimetic resulted in a substantial increase in MT-3 mRNA level in the adipocytes, the increase ranging from 400-fold with CoCl_2_ to 50-fold in the case of dimethyloxalylglycine (Fig. 3).

## Discussion

The present study has utilised PCR hypoxia-signalling pathway arrays to explore the integrated response of human adipocytes to low O_2_ tension. The arrays allow the expression of multiple genes to be simultaneously screened while using real-time PCR for the accurate quantification of changes in mRNA level. The majority of the genes on the arrays did not show any significant change in response to hypoxia. These genes included the insulin-sensitive glucose transporter, GLUT-4, consistent with our previous study on glucose transporters [21]. One particular gene, MT-3, a member of the metallothionein family of low mol. wt. (6000 *M*_r_), cysteine-rich proteins, was identified as being dramatically induced by hypoxia in human adipocytes. The increase in MT-3 mRNA level was rapid (by 60 min), as well as very substantial (up to 1600-fold in 24 h). No other gene on the arrays exhibited such a large response to hypoxia.

MT-3 is not the only member of the MT family expressed in white adipocytes. Previous studies have shown that the MT-1 and MT-2 genes are expressed by rodent adipocytes, with MT protein being secreted from rat fat cells [25], while expression of the MT-2A gene has been documented in human adipocytes [24]. In marked contrast to MT-3, MT-2A expression was minimally affected by hypoxia.

The key transcription factor in the signalling of the molecular response to low O_2_ tension is HIF-1 (hypoxia-inducible factor 1), which consists of α and β subunits [26–28]. The studies with the three hypoxia mimetics clearly implicate the HIF-1 signalling pathway in the hypoxia-induced expression of MT-3. Each mimetic leads to the stabilisation of HIF-1α, through inhibition of the prolyl hydroxylases which in the presence of O_2_ instigate the degradation of the transcription factor subunit [29,30]. Thus, MT-3 appears to be transcriptionally activated by HIF-1 in human adipocytes.

Preadipocytes, as well as adipocytes, exhibit a marked induction of MT-3 gene expression in response to low O_2_ tension. A major hypoxia-induced expression of MT-3 has previously been observed in human astrocytes [31]. MT-3, also known as growth inhibitory factor, has been considered largely as a brain-specific MT [31,32]. Studies with neurons have indicated that MT-3 is protective against toxic challenges and it is suggested that the hypoxia-induced expression in astrocytes protects the brain from hypoxic damage [31]. Protection against hypoxic stress may also be the primary function of MT-3 in adipocytes. MTs in general, particularly MT-1 and MT-2, have been implicated in zinc homeostasis, protection against metal toxicity and oxidative damage, and in stimulating angiogenesis [32].

The PCR arrays demonstrated that hypoxia also leads to increased expression of several other adipocyte genes. These include leptin, VEGF, angiopoietin-like protein 4, IL-6 and GLUT-1, and this is in agreement with our previous studies on human adipocytes using a candidate gene approach [20]. Hypoxia-stimulated expression of these genes has also been demonstrated in murine adipocytes [13,15]. The array studies further show that IGF-2 gene expression in human adipocytes is stimulated by hypoxia, as is the glycolytic enzyme, glucose phosphate isomerase. The increase in expression of glucose phosphate isomerase is indicative of higher rates of glycolysis when O_2_ is limited. This would be consistent with our recent report that hypoxia leads to increased synthesis of GLUT-1, together with elevated glucose uptake, by human adipocytes [21].

The expression of several genes on the arrays was downregulated by hypoxia, and interestingly these include catalase and glutathione peroxidase 1, both of which are associated with the response to oxidative stress and particularly the breakdown of hydrogen peroxide. The expression of caspase 1, an enzyme involved in apoptosis, was also inhibited, as was the mitochondrial carrier protein, uncoupling protein 2.

MT-3 mRNA was detected here in human adipose tissue, as well as in adipocytes in culture. Although the WAT samples were obtained from obese subjects, it is unclear whether the presence of MT-3 mRNA is indicative of hypoxia within the tissue in human obesity. Direct evidence for hypoxia in adipose tissue in obesity has recently been obtained in rodents [12–14], and a growing number of genes have been shown to be hypoxia-sensitive in adipocytes, both rodent and human. Several of these genes are linked with the inflammatory response and angiogenesis, consistent with the original hypothesis that inflammation in adipose tissue in obesity is a consequence of localised hypoxia within the tissue [3].

## Figures and Tables

**Table 1:** Genes on hypoxia-signalling pathway PCR arrays whose expression is up- or down-regulated in human adipocytes by hypoxia

Gene	Protein	Change with hypoxia (fold)	*P* value
*Upregulated: 24 h*			
MT3	Metallothionein-3	614.6	<0.001
LEP	Leptin	20.9	<0.001
VEGF	Vascular endothelial growth factor	20.7	<0.001
SLC2A1	GLUT-1 (facilitative glucose transporter 1)	12.4	<0.001
ANGPTL4	Angiopoietin-like protein 4	10.5	<0.001
BHLHB2	Basic helix-loop-helix domain containing, class B,2	3.94	<0.001
CYGB	Cytoglobin	3.64	<0.001
IL6	Interleukin-6	2.90	<0.01
IGF2	Insulin-like growth factor 2	2.67	<0.02
GAPDH	Glyceraldehyde-3-phosphate dehydrogenase	2.67	<0.001
GPI	Glucose phosphate isomerase	2.22	<0.001
HIF3A	Hypoxia inducible factor 3, α-subunit	2.01	<0.001
			
*Downregulated: 24 h*			
CASP1	Caspase 1, apoptosis-related cysteine protease	−4.44	<0.001
HIF1A	Hypoxia inducible factor 1, α-subunit	−4.11	<0.001
EPAS1	Endothelial PAS domain protein 1	−3.17	<0.002
SSSCA1	Sjogren’s syndrome/sclerodoma autoantigen 1	−3.14	<0.02
GPX1	Glutathione peroxidase 1	−3.07	<0.05
AGPAT2	1-Acylglycerol-3-phosphate *O*-acyltransferase 2	−2.55	<0.002
CAT	Catalase	−2.53	<0.02
UCP2	Uncoupling protein 2	−2.40	<0.001
PSMB3	Proteasome subunit, beta type, 3	−2.15	<0.002
			
*Upregulated: 4 h*			
MT3	Metallothionein-3	121.0	<0.05
SLC2A1	GLUT-1 (facilitative glucose transporter 1)	4.03	<0.002
VEGF	Vascular endothelial growth factor	3.72	<0.001
RPL28	Ribosomal protein L28	3.20	<0.05
ADM	Adrenomedullin	3.12	<0.05
BHLHB2	Basic helix-loop-helix domain containing, class B,2	2.45	<0.02
LEP	Leptin	2.19	<0.05
HK2	Hexokinase 2	2.01	<0.05

Human adipocytes differentiated from preadipocytes in culture were incubated under normoxia (21% O_2_) or hypoxia (1% O_2_) for 4 or 24 h. Hypoxia-signalling pathway PCR arrays (Superarray) were used to screen those genes whose expression is hypoxia-sensitive. The genes listed are those where there was a statistically significant (*P* < 0.05) change of at least 2-fold. The fold changes are derived from 4 groups of normoxic and hypoxic adipocytes at 24 h and 3 groups at 4 h.
